# Bryophytes Occurrences Dataset Based On SYKO Herbarium Moss Collection

**DOI:** 10.3897/BDJ.8.e57942

**Published:** 2020-10-13

**Authors:** Galina Zheleznova, Tatyana Shubina, Mikhail Rubtsov, Galina Litvinenko, Ivan Chadin

**Affiliations:** 1 Institute of Biology of Komi Science Centre of the Ural Branch of the Russian Academy of Sciences, Syktyvkar, Russia Institute of Biology of Komi Science Centre of the Ural Branch of the Russian Academy of Sciences Syktyvkar Russia

**Keywords:** Bryophyta, Marchantiophyta, GBIF, data paper, preserved specimen, herbarium labels digitisation, Komi Republic, Russia

## Abstract

**Background:**

The dataset with 49,726 bryophytes occurrences (49,261 moss occurrences and 465 liverworts occurrences), located predominantly on the territory European north-east Russia, is described in this data paper. The dataset was based on the digitised moss labels from the Institute of Biology of Komi Scientific Сenter of the Ural Branch of the Russian Academy of Sciences herbarium (SYKO). The information from the labels was recognised, cleaned and brought into compliance with the Darwin Core. More than 99.9% of occurrences were georeferenced with a precision of at least 3 km. For each occurrence, the original label image URL was given. The dataset contains occurrences of 539 moss and liverworts taxa (species and lower ranks) belonging to 190 genera and 75 families.

**New information:**

Information about 49,726 bryophytes occurrences was published in GBIF. The dataset was based on label data of 94% of SYKO herbarium moss collection specimens. Most of the occurrences were described with the following fields: occurrenceID, institutionID, collectionCode, catalogNumber, basisOfRecord, scientificName, taxonRank, kingdom, phylum, class, order, family, genus, recordedBy, identifiedBy, associatedMedia, day, month, year, country, countryCode, decimalLatitude, decimalLongitude, geodeticDatum, coordinateUncertaintyInMetres, georeferencedBy.

## Introduction

The herbarium of Institute of Biology of Komi Science Centre of the Ural Branch of the Russian Academy of Sciences (SYKO) is one of the largest herbaria on European north-ast Russia with more than 309,800 specimens. It was established by the famous Russian botanist A. Tolmachev in Syktyvkar city in 1941. There are five subdivisions in SYKO Herbarium: vascular plants (205,000 specimens), bryophytes (58,184 specimens), algae (17,420 specimens), lichens (26,000 specimens) and fungi (3,207 specimens).

The next largest SYKO herbarium subdivision after the vascular plant's subdivision is the bryophytes' one organised by I. Kildjushevskij in 1969. There are two collections in this subdivision: moss collection and liverworts collection. These collections were based on the specimens collected during the Komi Republic vegetation exploration in 1933-1968. It should be noted that there are some liverworts samples in the moss collection. These liverworts samples are stored, not in the form of separate storage units, but as a mixture of specimens containing several species collected at one point.

There are exsiccata from other herbaria (LE, KPABG) in this SYKO subdivision. The exsiccata originated from the territories of European Russia, Caucasus, Western and Southern Siberia, Russian Far East, Ukraine, the Republic of Kazakhstan, the Republic of Azerbaijan, the Republic of Tajikistan, Mongolia and USA (Alaska). The exsiccata labels were not planned for digitising as it was thought better for the community to have this information published by the original herbaria.

The bryophytes' subdivision of SYKO herbarium is an important reference source for the study of the moss flora of the European north-east Russia and, in particular, for such a large region (416,774 km²) as the Komi Republic (Zheleznova 1994, Degteva et al. 2001, Shubina and Zheleznova 2002).

All rare and protected moss species (43 species, 289 occurrences) are presented as specimens in the bryophyte collection. These specimens were used for preparation of three editions of the Komi Republic Red Data Book (Taskaev 1998, Taskaev 2009, Degteva 2019). The herbarium SYKO bryophyte collection is increasing by approximately 700 curation units annually as a result of fieldworks.

## General description

### Purpose

This data paper was written in concordance with concept described in works of Vishwas Chavan and Lyubomir Penev (Chavan and Penev 2011, Penev et al. 2017). A data paper publication for any given dataset strongly influences the dataset quality, so the purpose of this paper was to describe the herbarium-based bryophytes occurrences dataset published in GBIF ([Bibr B6071123][Bibr B6071811]).

## Project description

### Title

European north-east Russia moss occurrence data mobilisation on the basis of the SYKO herbarium moss collection

### Personnel

Ivan Chadin (team leader, programmer), Tatyana Shubina (bryologist, data entering and revision), Galina Zheleznova (bryologist, collection curator, data entering and revision), Galina Litvinenko (label image capturing, data entering), Mikhail Rubtsov (label georeferencing).

### Study area description

The aim of the project was to digitise at least 8,000 labels from the SYKO herbarium moss collection. As a result of the project, 14,000 labels were digitised and the final version (1.5) of the dataset, published in GBIF, contained 14,871 moss occurrences. The project team consisted of five people, all of them being the authors of this work.

### Funding

The herbarium labels for this dataset were mobilised with support from The Global Biodiversity Information Facility Secretariat (GBIFS). Project ID: Russia2019_04. Project web-page: https://www.gbif.org/project/5ZsAifyI6z0OguyoNTFIIu/mobilizing-moss-occurrences-from-the-komi-science-centre-herbarium. Duration: 01.02.2019-30.09.2019.

## Sampling methods

### Study extent

The bryophytes subdivision of SYKO is divided into two collections: mosses and liverworts. We have not digitised the labels of the liverworts collection at this moment. However, some occurrences of liverworts were added in the dataset as a result of keeping them simultaneously in one specimen packet with mosses. The labels of the liverworts collection are planned for digitisation in the near future.

According to the SYKO bryophytes subdivision register (maintained manually since 1969), there were 58,184 specimens (45,198 mosses and 12,986 liverworts) at the beginning of August 2020. The label data of 42,698 unique moss samples (94 percent of moss collection) have been digitised to that date. The 1,697 moss storage units have duplicates (specimens that have the same label data as original specimens). We stored these duplicates in the main collection and used them for exchange with other institutions. The duplicates were not used for the described occurrence dataset preparation. The collection of mosses is characterised by the frequent presence of more than one species in one specimen (from 1 to 9 species per specimen, 1.2 on average).

Some parts of the digitised labels were excluded from the described dataset. A total of 2,754 labels were used for updating the dataset “Moss occurrences in Yugyd Va National Park, Subpolar and Northern Urals, European North-East Russia” published earlier (Zheleznova et al. 2020a). The images for 3,452 occurrences published earlier were added in the field “associatedMedia” for the Yugyd Va dataset.

Thus, the dataset, described in this paper, was based on 39,916 of 42,698 digitised moss labels which allowed us to publish 49,726 occurrences (Zheleznova et al. 2020b).

### Sampling description

Bryophytes herbarium samples were collected during two main types of fieldwork: floristic explorations and vegetation studies. Field samples were separated into storage specimens during the species identification in a way that, in each specimen, there was a minimum number of bryophyte species. Two label copies are generated for each sample. One copy of the label was fixed on a bag with a dried moss sample, the second was stored in a separate storage for labels (library card catalogue cabinet was used). The labels and the moss specimens themselves were arranged in alphabetical order of species names. Each moss sample was assigned a catalogue number. The catalogue numbers have been increasing since the time of the organisation of the bryophytes subdivision in the SYKO herbarium. Information about the label catalogue number, date of collection, name of the collection place, species name, field number and habitat were entered in the register.

The labels from label storage were used for digitisation. The label images were obtained with a digital camera. Images were uploaded to the server and their filenames to the label database. The database web interface, written specifically for this project, was used for manual label data recognition and interpretation. The following minimum set of data were deciphered (in Darwin Core terms): scientificName, recordedBy, identifiedBy, day, month, year, catalogNumber, decimalLatitude, decimalLongitude.

The digitisation of most of the moss collection labels showed that the names of 139 collectors were on the labels and 38 botanists were engaged in species identification. The most productive collector and botanist who was principallyengaged in species identification was one person — G. Zheleznova (Tables 1, 2).

### Quality control

**Species identification.** The species were identified by bryologists from the Institute of Biology of Komi Scientific Centre of the Ural Branch of the Russian Academy of Sciences. The correctness of species identification and confirmation for many taxa was carried out by well-known taxonomy specialists: R. Schljakov (378 identifications), A. Abramova (246 identifications), L. Savicz (110 identifications), M. Ignatov (58 identifications), O. Afonina (52 identifications), E. Ignatova (49 identifications), I. Czernjadieva (16 identifications), Z. Smirnova (15 identifications), V. Fedosov (11 identifications), I. Abramov (10 identifications) and A. Maksimov (2 identifications).

Some moss samples were sent for critical review to other herbaria. For example, the specimens with genera *Pohlia* Hedw., *Stereodon* (Brid.) Mitt., *Hypnum* Hedw., *Cratoneuron* (Sull.) Spruce, *Hygrohypnum* Lindb., *Tortella* (Müll.Hal.) Limpr., *Pseudoleskeella* Ignatov & Ignatova were sent to the Komarov Botanical Institute herbarium (LE), *Grimmia* Hedw., *Schistidium* Bruch & Schimp., *Philonotis* Brid., *Bryum* Hedw., *Bucklandiella* Roiv., *Polytrichum* Hedw., *Lescuraea* Bruch & Schimp., *Sciuro-hypnum* (Hampe) Hampe to The Tsytsin Main Moscow Botanical Garden of Academy of Sciences herbarium (MHA), genera *Encalypta* Hedw., *Seligeria* Bruch & Schimp. to the herbarium of Moscow University (MW).

**Label images quality.** Each image of the label was checked for readability by operators who deciphered label data. Images that were out of focus or had extraneous objects in the frame were deleted from the database. It was possible to recapture bad label images only if the catalogue number of the label was detectable on discarded images. In other cases (about 6% of the total number of labels in the moss collection), the second round of label image capturing will be performed later (after forming a list of missing labels with the help of the label register).

**Check of georeferencing.** Occurrences locations were added to a map with the OpenStreetMap layer and with Russian regions borders polygon layers in QGIS software (Open Source Geospatial Foundation Project 2020). The names of regions were assigned to each occurrence with the help of “Point Sampling Tool” QGIS plugin. The occurrences located outwith the land border of any Russia region and occurrences located far from the borders of Komi Republic were subject to verification.

**Text recognition quality**. All label data recognised by operators were checked visually for each label image. Special boolean-like fields were added to the database table with main label information: the check was carried out (yes / no), data clarification is required (yes / no). The label data needing to be checked were divided in two groups: 1) the collection date and catalogue number, 2) names of taxa indicated on the label and the names of people who collected the sample and who identified the species.

Additional verification of collection dates and collectors names was carried out during labels georeferencing. It is known that one collector could not be in all points located more than several kilometres from each over during the same day. After the main array of labels digitising and recognition, it became possible to compare the series of labels to identify and correct obvious errors that were made, not only during image data recognition, but also errors that were made by laboratory technicians during manual filling out of label blanks. In the latter case, corrected information was added in the database and the label was marked for replacement in the near future.

**Taxonomy validation.** Verbatim taxon names indicated on labels, in many cases, were out of date and not valid. In our case, only professional bryologists were the operators for taxon name recognition, so verbatim names were corrected on the fly during data entering in the database. The next step of taxon name checking was normalising species names against the GBIF backbone (https://www.gbif.org/tools/species-lookup). The GBIF backbone normalised species names and higher taxonomy were updated manually by our bryologists to bring the taxon name usage in concordance with the latest moss checklists (Ignatov et al. 2006, Hodgetts et al. 2020).

**Dataset validation.** The publication-ready Darwin Core compliant dataset was generated as a csv-file by Python script which included SQL queries to the database. This file was checked for errors manually with the data filtering function of spreadsheet software and automatically with the GBIF Data Validator service (https://www.gbif.org/tools/data-validator).

### Step description

1. The database and web application for database administration were created with MariaDB (https://mariadb.com) and Django framework (https://www.djangoproject.com).

2. Batch of labels images were captured per box (drawer) of the labels' catalogue with strict adherence to the labels order in each box. Labels in boxes are kept in alphabetical order of taxon names. Labels of samples collected on the same dates by the same collectors were often grouped within every box. Label images captured in the order they were kept allowed us to significantly simplify the data recognition process for operators. Images were taken with a photo camera with a minimum frame size of 4000 × 3000 pixels.

3. Batch of labels images up to several thousands JPEG files were processed simultaneously. Each image was cropped to remove most of the background so the image size became approximately 2000 × 1500 pixels. The white balance of all images was automatically adjusted with Fred Weinhaus ‘autowhite’ script for ImageMagick software (http://www.fmwconcepts.com/imagemagick/autowhite).

4. Cropped images were uploaded to the server and their file path names were added in label database.

5. An operator decrypted the label data with a web application. Different web forms for different types of data were used: entering catalogue number and collection date; entering the names of taxa; entering the names of the collectors and persons who carried out the identification of taxa; input of geographic coordinates. Dates were entered as three separate numbers: day, month and year. This format of dates storage allowed the processing of labels with omitted days or month in collection date. Qualified bryologists entered the names of taxa, the names of the collectors and the persons who identified the species of mosses. Georeferencing of labels was performed by an engineer with cartographic skills. In some cases, for a more accurate determination of coordinates, it was possible to question the collector of the sample.

6. All entered data (excluding geographic coordinates) were checked with special forms in the web application. Label images were compared with entered data and errors were corrected simultaneously or marked for correction later.

## Geographic coverage

### Description

**Figure 1.**
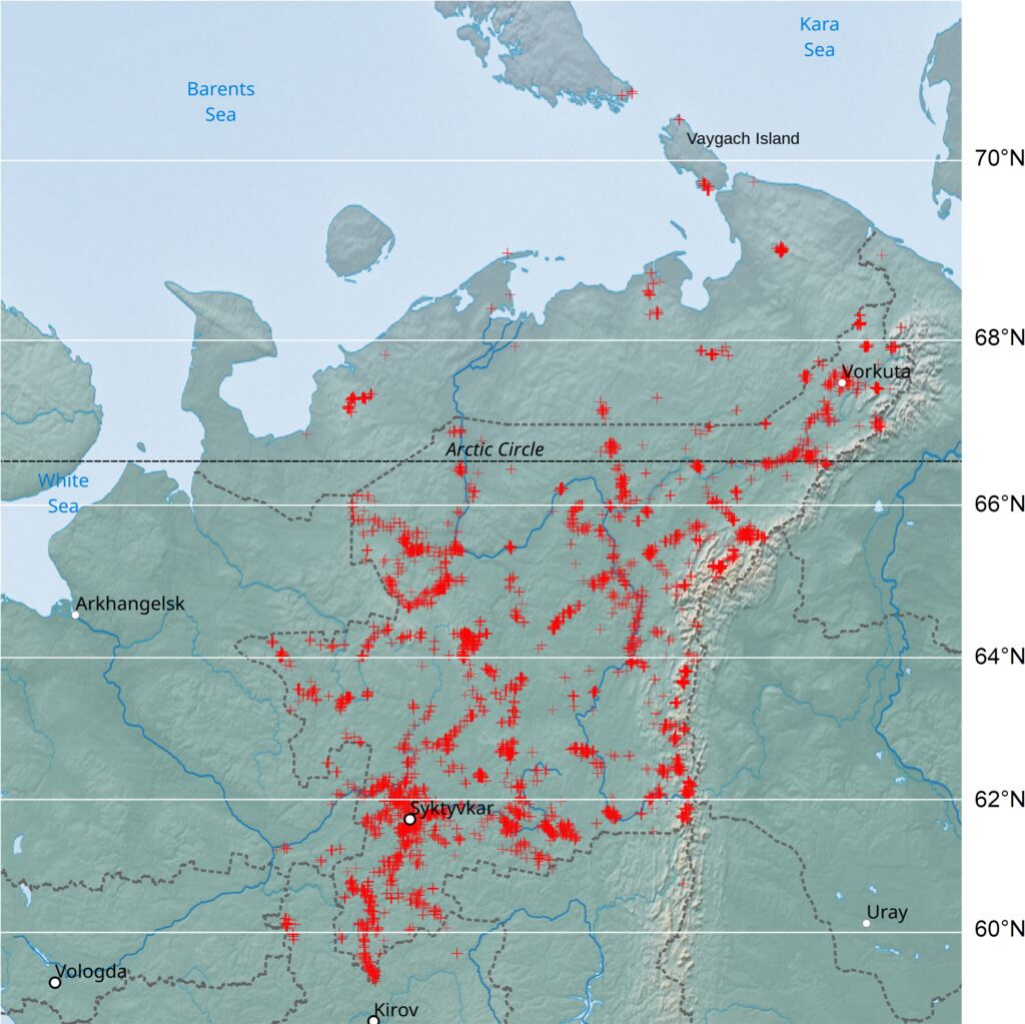
The location (red crosses) of the most occurrences of the dataset.

Most of the dataset occurrences were located in the territory of European north-east Russia. Only one occurrence was located far from this region on the Kamchatka Peninsula (55.72222°N, 160.3714°E). The polygon with the shortest perimeter that encloses most of the occurrences (the convex hull) was approximately 820,000 square kilometres (Fig. 1). In total, the dataset contained 3,918 collection sites for bryophyte specimens with unique geographic coordinates. The point with the largest number of occurrences (1564) was located on Vaygach Island (69.75°N, 59.82°E).

Most of the published occurrences were located in the territory of the Komi Republic (86% of all occurrences) and the Nenets Autonomous district (12%). The remaining occurrences (2%) were collected mainly in the territory of seven the Komi Republic neighbouring regions (Table 3).

### Coordinates

59.23 and 70.72 Latitude; 68.63 and 46.55 Longitude.

## Taxonomic coverage

### Description

This dataset contained 47,955 occurrences of 480 moss taxa with rank of species, subspecies, varieties and 465 occurrences of 59 liverworts taxa of the same ranks (Zheleznova et al. 2020b, Zheleznova et al. 2020c). The species names used were determined according mostly to the ‘Check-list of mosses of East Europe and North Asia’ (Ignatov et al. 2006) and ‘An annotated checklist of bryophytes of Europe, Macaronesia and Cyprus‘ (Hodgetts et al. 2020). The first one (Ignatov et al. 2006) was the primary source if checklists contradicted.

The specimen and labels catalogue were re-arranged if valid names changed. Considering the latest sources (Ignatov and Milyutina 2007, Hodgetts et al. 2020), all samples identified earlier as Brachythecium curtum (Lindb.) Limpr., B. oedipodium (Mitt.) A. Jaeger, B. starkei var. curtum (Lindb.) Warnst., Sciuro-hypnum oedipodium (Mitt.) Ignatov & Huttunen were assigned to Sciuro-hypnum curtum (Lindb.) Ignatov. There were 520 samples of Sphagnum magellanicum Bridel, 1798 in the herbarium and all of them need to be revised in accordance with Hassel et al. 2018 (Hassel et al. 2018).

The dataset contained occurrences of five Bryophyta classes and two classes of Marchantiophyta. Most abundant classes were Bryopsida, Polytrichopsida and Sphagnopsida which represented more than 98% of all published occurrences (Table 4).

Most of the moss species sampled in the herbarium were sufficiently abundant to be collected in hundreds and sometimes thousands of samples. Seven moss species were represented in more than 1000 occurrences (Table 5). These are the most widespread mosses in the European north-east Russia. They account for 24% of all moss finds in the published dataset.

**Figure 2.**
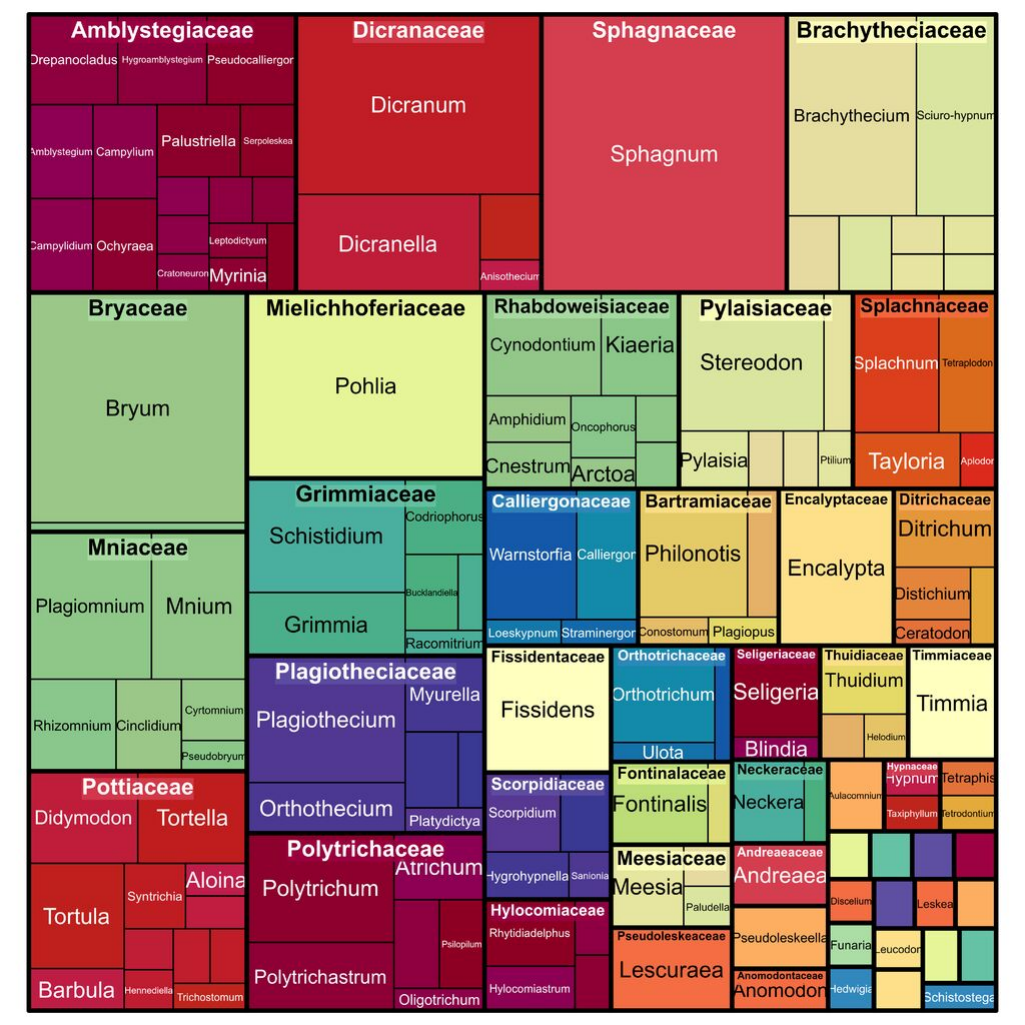
Taxonomic diversity of moss families in the dataset. The figure was prepared with the “treemap” package in R (Tennekes 2017).

**Figure 3.**
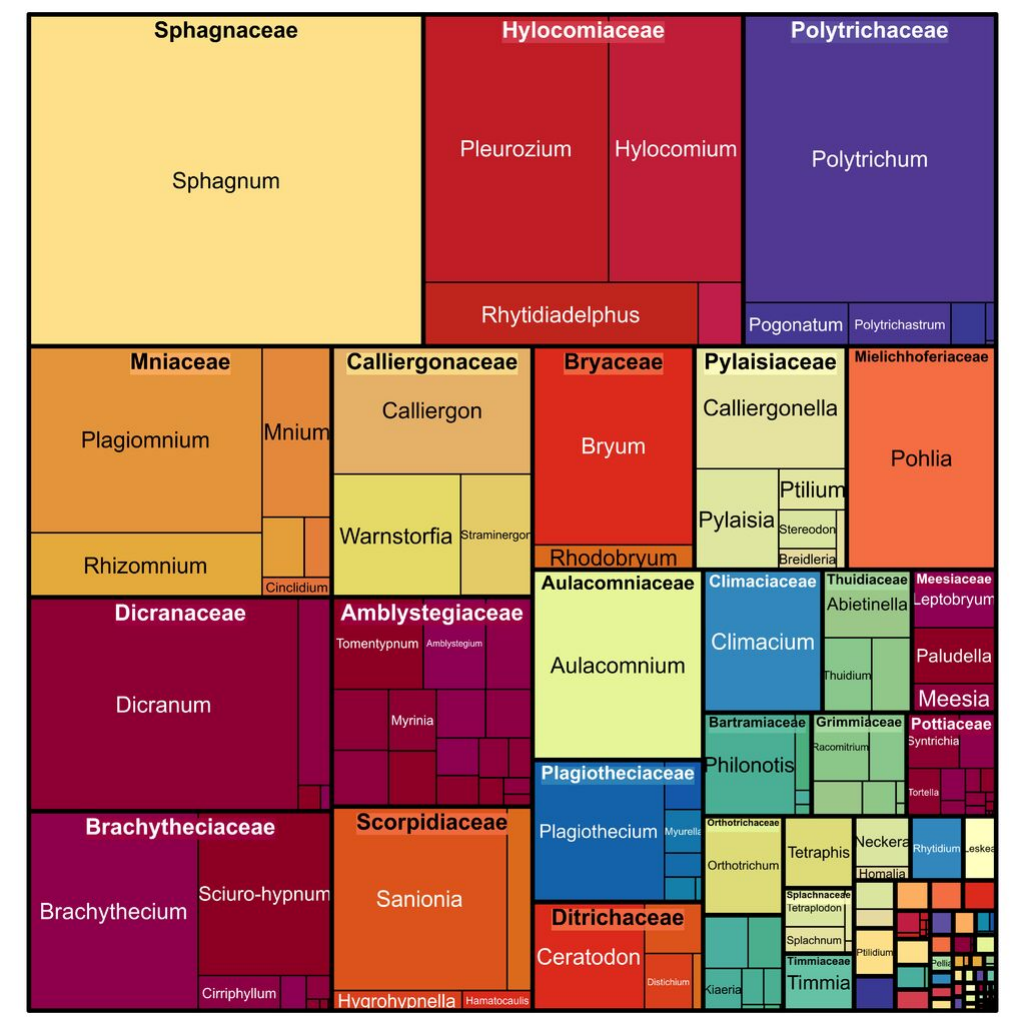
Taxonomic distribution of occurrences amongst moss families in the dataset. The figure was prepared with the “treemap” package in R (Tennekes 2017).

The most numerous Bryophyta families in terms of the occurrences were the following: Sphagnaceae, Hylocomiaceae, Polytrichaceae, Mniaceae, Dicranaceae, Brachytheciaceae, Calliergonaceae, Amblystegiaceae, Scorpidiaceae, Bryaceae (Table 6 Figs 2, 3).

### Taxa included

**Table:** 

Rank	Scientific Name	Common Name
kingdom	Plantae	Plants
phylum	Bryophyta	Mosses
phylum	Marchantiophyta	Liverworts
class	Andreaeopsida	
class	Bryopsida	
class	Jungermanniopsida	
class	Marchantiopsida	
class	Polytrichopsida	
class	Sphagnopsida	
class	Tetraphidopsida	
order	Andreaeales	
order	Blasiales	
order	Bryales	
order	Buxbaumiales	
order	Catoscopiales	
order	Dicranales	
order	Encalyptales	
order	Funariales	
order	Grimmiales	
order	Hedwigiales	
order	Hypnales	
order	Jungermanniales	
order	Marchantiales	
order	Metzgeriales	
order	Orthotrichales	
order	Pelliales	
order	Polytrichales	
order	Porellales	
order	Pottiales	
order	Ptilidiales	
order	Sphagnales	
order	Splachnales	
order	Tetraphidales	
order	Timmiales	
family	Amblystegiaceae	
family	Anastrophyllaceae	
family	Andreaeaceae	
family	Aneuraceae	
family	Anomodontaceae	
family	Arnelliaceae	
family	Aulacomniaceae	
family	Aytoniaceae	
family	Bartramiaceae	
family	Blasiaceae	
family	Blepharostomataceae	
family	Brachytheciaceae	
family	Bruchiaceae	
family	Bryaceae	
family	Buxbaumiaceae	
family	Calliergonaceae	
family	Calypogeiaceae	
family	Catoscopiaceae	
family	Cephaloziaceae	
family	Cephaloziellaceae	
family	Climaciaceae	
family	Conocephalaceae	
family	Dicranaceae	
family	Disceliaceae	
family	Ditrichaceae	
family	Encalyptaceae	
family	Fissidentaceae	
family	Fontinalaceae	
family	Funariaceae	
family	Grimmiaceae	
family	Gymnomitriaceae	
family	Harpanthaceae	
family	Hedwigiaceae	
family	Heterocladiaceae	
family	Hylocomiaceae	
family	Hypnaceae	
family	Jungermanniaceae	
family	Lepidoziaceae	
family	Leskeaceae	
family	Leucobryaceae	
family	Leucodontaceae	
family	Lophocoleaceae	
family	Lophoziaceae	
family	Marchantiaceae	
family	Meesiaceae	
family	Metzgeriaceae	
family	Mielichhoferiaceae	
family	Mniaceae	
family	Myliaceae	
family	Neckeraceae	
family	Orthotrichaceae	
family	Pelliaceae	
family	Plagiochilaceae	
family	Plagiotheciaceae	
family	Polytrichaceae	
family	Pottiaceae	
family	Pseudoleskeaceae	
family	Pseudoleskeellaceae	
family	Pterigynandraceae	
family	Ptilidiaceae	
family	Pylaisiaceae	
family	Pylaisiadelphaceae	
family	Radulaceae	
family	Rhabdoweisiaceae	
family	Rhytidiaceae	
family	Ricciaceae	
family	Scapaniaceae	
family	Schistostegaceae	
family	Scorpidiaceae	
family	Seligeriaceae	
family	Sphagnaceae	
family	Splachnaceae	
family	Tetraphidaceae	
family	Thuidiaceae	
family	Timmiaceae	

## Temporal coverage

**Data range:** 1933-7-14 – 2019-10-09.

### Notes

The earliest moss samples were collected by A. Shennikov in 1933 near Adak-Shelya village (the Inta District), by A. Lashenkova in 1934, N. Dylis and A. Zueva in 1935 in Syktyvkar suburb. Systematic collection of bryophytes began in 1969 and continues to the present.

## Collection data

### Collection name

Moss collection of herbarium of Institute of Biology of Komi Science Сenter of the Ural Branch of the Russian Academy of Sciences

### Collection identifier

SYKO

### Specimen preservation method

Dried and pressed

### Curatorial unit

58184

## Usage rights

### Use license

Open Data Commons Attribution License

## Data resources

### Data package title

SYKO Herbarium Moss Collection. Occurrences and Checklist.

### Number of data sets

2

### Data set 1.

#### Data set name

SYKO Herbarium Moss Collection

#### Data format

Darwin Core Archive

#### Number of columns

29

#### Character set

UTF-8

#### Download URL

https://www.gbif.org/dataset/3412de46-ed80-42c1-9e7b-42a1e040e66e

#### 

**Data set 1.** 

Column label	Column description
occurrenceID	An identifier for the Occurrence
institutionID	An identifier for the institution having custody of the object(s) or information referred to in the record
collectionCode	The name, acronym, coden or initialism identifying the collection or dataset from which the record was derived.
catalogNumber	An identifier (preferably unique) for the record within the dataset or collection.
basisOfRecord	The specific nature of the data record.
scientificName	The full scientific name, with authorship and date information, if known. When forming part of an Identification, this should be the name in the lowest level taxonomic rank that can be determined. This term should not contain identification qualifications, but should instead be supplied in the IdentificationQualifier term.
taxonRank	The taxonomic rank of the most specific name in the scientificName.
kingdom	The full scientific name of the kingdom in which the taxon is classified.
phylum	The full scientific name of the phylum or division in which the taxon is classified.
class	The full scientific name of the class in which the taxon is classified.
order	The full scientific name of the order in which the taxon is classified.
family	The full scientific name of the family in which the taxon is classified.
genus	The full scientific name of the genus in which the taxon is classified.
recordedBy	A list (concatenated and separated) of names of people, groups or organisations responsible for recording the original Occurrence. The primary collector or observer, especially the one who applies a personal identifier (recordNumber), should be listed first.
identifiedBy	A list (concatenated and separated) of names of people, groups or organisations who assigned the Taxon to the subject.
associatedMedia	A list (concatenated and separated) of identifiers (publication, global unique identifier, URI) of media associated with the Occurrence
day	The integer day in which the sample was collected
month	The ordinal month in which the sample was collected
year	The ordinal year in which the sample was collected
country	The name of the country or major administrative unit in which the Location occurs.
countryCode	The standard code for the country in which the Location occurs.
decimalLatitude	The geographic latitude (in decimal degrees, using the spatial reference system given in geodeticDatum) of the geographic centre of a Location.
decimalLongitude	The geographic longitude (in decimal degrees, using the spatial reference system given in geodeticDatum) of the geographic centre of a Location.
geodeticDatum	The ellipsoid, geodetic datum or spatial reference system (SRS) upon which the geographic coordinates given in decimalLatitude and decimalLongitude are based.
coordinateUncertaintyInMetres	The horizontal distance (in metres) from the given decimalLatitude and decimalLongitude describing the smallest circle containing the whole of the Location. Leave the value empty if the uncertainty is unknown, cannot be estimated or is not applicable (because there are no coordinates). Zero is not a valid value for this term.
georeferencedBy	A list (concatenated and separated) of names of people, groups or organisations who determined the georeference (spatial representation) for the Location.
specificEpithet	The name of the first or species epithet of the scientificName.
infraspecificEpithet	The name of the lowest or terminal infraspecific epithet of the scientificName, excluding any rank designation.
eventDate	The date-time or interval during which an Event occurred. For occurrences, this is the date-time when the event was recorded. Not suitable for a time in a geological context.

### Data set 2.

#### Data set name

Bryophyte Checklist of SYKO Herbarium

#### Data format

Darwin Core Archive

#### Number of columns

12

#### Character set

UTF-8

#### Download URL

https://www.gbif.org/dataset/d5a07901-27f3-4100-99fb-e393097f6233

#### 

**Data set 2.** 

Column label	Column description
taxonID	An identifier for the set of taxon information (data associated with the Taxon class).
scientificName	An identifier for the nomenclatural (not taxonomic) details of a scientific name.
taxonRank	The taxonomic rank of the most specific name in the scientificName.
kingdom	The full scientific name of the kingdom in which the taxon is classified.
phylum	The full scientific name of the phylum or division in which the taxon is classified.
class	The full scientific name of the class in which the taxon is classified.
order	The full scientific name of the order in which the taxon is classified.
family	The full scientific name of the family in which the taxon is classified.
genus	The full scientific name of the genus in which the taxon is classified.
nameAccordingTo	An identifier for the source in which the specific taxon concept circumscription is defined or implied.
specificEpithet	The name of the first or species epithet of the scientificName.
infraspecificEpithet	The name of the lowest or terminal infraspecific epithet of the scientificName, excluding any rank designation.

## Figures and Tables

**Table 1.** Top ten collectors for the SYKO herbarium moss collection dataset.

**Collector’s Name**	**Occurrences**
G. Zheleznova	19448
M. Dulin	7276
I. Kildjushevskij	5722
A. Kustysheva	2493
B. Teterjuk	2298
E. Kuljugina	1913
T. Shubina	1824
V. Frolova	1747
S. Degteva	1374
A. Lashhenkova	1342

**Table 2.** Names of botanists who identified species for most of the occurrences of the SYKO herbarium moss collection dataset.

**Botanist’s Name**	**Occurrences**
G. Zheleznova	27468
T. Shubina	11104
I. Kildjushevskij	7408

**Table 3.** Distribution of bryophyte occurrences amongst Russian Federation regions.

**Region**	**Occurrences**	**Percentage**
Komi Republic	42796	86
Nenets Autonomous Okrug (including Vaygach Island)	6003	12
Kirov Oblast	565	1
Arkhangelsk Oblast	140	< 1
Vologda Oblast	106	< 1
Yamalo-Nenets Autonomous Okrug	94	< 1
Sverdlovsk Oblast	18	< 1
Perm Krai	3	< 1
Kamchatka Krai	1	< 1

**Table 4.** Distribution of occurrences amongst classes of phylum Bryophyta and phylum Marchantiophyta.

**Taxon**	**Occurrences**
** Bryophyta **	
Bryopsida	37861
Polytrichopsida	4329
Sphagnopsida	6756
Tetraphidopsida	263
Andreaeopsida	52
** Marchantiophyta **	
Marchantiopsida	100
Jungermanniopsida	365

**Table 5.** Moss species with more than 1000 occurrences.

**Species**	**Occurrenses**
*Pleurozium schreberi* (Willd. ex Brid.) Mitt.	2560
*Hylocomium splendens* (Hedw.) Schimp.	1863
*Sanionia uncinata* (Hedw.) Loeske	1666
*Aulacomnium palustre* (Hedw.) Schwägr.	1317
*Polytrichum commune* Hedw.	1250
*Plagiomnium ellipticum* (Brid.) T.J.Kop.	1208
*Polytrichum juniperinum* Hedw.	1080

**Table 6.** Top ten families with most numerous occurrences.

**Moss family**	**Occurrences**	**Percentage of the total occurrence number**
Sphagnaceae	6756	14
Hylocomiaceae	5478	11
Polytrichaceae	4329	9
Mniaceae	3924	8
Dicranaceae	3344	7
Brachytheciaceae	3116	6
Calliergonaceae	2600	5
Amblystegiaceae	2170	4
Scorpidiaceae	2116	4
Bryaceae	1866	4
